# Meeting of the American Medical Association, at Baltimore, May 2d, 1848

**Published:** 1848-06

**Authors:** 


ART. II.—*Meeting of the American Medical Association, at Baltimore, May 2d,* 1848.

*First day.*—The association met in the Universalist church at 11 o'clock A. M. and was called to order by the President, Dr. N. Chapman. Most of this day was consumed in preliminary arrangements and in preparing for the election of officers. Two ballotings had resulted in the election of only three officers, when the association adjourned.

*Second day.*—The association was called to order at 9 o'clock by Dr. Jonathan Knight, Vice President.

The second ballot, had on Tuesday evening, was, on motion of Dr. Hays, of Philadelphia, set aside, as many of the members had voted under a misapprehension as to who were the nominees.

Dr. Jackson, who had been elected one of the Vice Presidents, Dr. Hays, who had been elected Treasurer, and Dr. Stille who was elected Secretary, each resigned the post assigned him, and a committee consisting of one from each State, was appointed to report officers for the Association. The committee retired to perform the duty assigned them, and

The Secretary proceeded to read a communication from the College of Pharmacy, of New York city, relative to the immense quantity of spurious and adulterated drugs which are now imported into this country, and asking the aid of the Association in bringing the matter before Congress in such a manner as to promote the adoption of some remedial law.

Dr. Edwards, a member of the House of Representatives from Ohio, and chairman of a special committee to whom the subject of the importation of spurious and adulterated drugs has been referred in the House, was then introduced, and stated a number of facts relative to the alarming and pernicious extent to which the most important and useful articles in the materia medica are imported into this country, in a state so adulterated, as to render them totally unfit for medicinal purposes.

The thanks of the Association were returned to Dr Edwards, and the Chair authorized to appoint a committee to memorialize Congress on the subject.

The Committee appointed to nominate officers, reported the following which were unanimously confirmed:

PRESIDENT—Dr. A. H. Stevens, of New York.

VICE-PRESIDENTS—Dr. Samuel Jackson, of Philadelphia; Dr. John C. Warren, of Boston; Dr. Paul F. Eve, of Augusta, Ga. and Dr. Wm. L. Awl, of Columbus, Ohio.

SECRETARIES—Dr. Alfred Stille, of Philadelphia; Dr. H. J. Bowditch, of Boston.

TREASURER.—Dr. Isaac Hays, of Philadelphia.

The President, on taking his seat, acknowledged the honor which had been conferred on him.

Dr. Harvey Lindsly, Chairman of the Committee on Obstetrics, read a report on the subject. The report was mainly devoted to the consideration of chloroform, or ether, during parturition, to which, under certain restrictions, the report was decidedly favourable. The report was accepted and ordered to be printed, *with the understanding that the Association thereby expressed no opinion for, or against the opinions advanced in it.*

Dr. Geo. W. Norris, Chairman of the Committee on Surgery, read to the Association an able report on the subject, relative to improvements made in the practice of surgery during the past year. The report of the Committee on another branch, namely, the use of ether and chloroform in alleviating the pain of the patient during surgical operations, was read by Dr. Isaac Parish, of Philadelphia. The report did not decide on the relative merits either of chloroform or ether, but was decidedly favourable to the use of them in difficult and painful cases.

The Chair announced the following as the Committee to memorialize Congress on the subject of the importation of adulterated and spurious drugs; viz:—Drs. Sprague, of Mass., Cox, of Md., Francis, of N. York, Hustun, of Pa., and Carr, of Vermont.

The Convention adjourned until 4 o'clock.

EVENING SESSION.

On motion of Dr. Stewart, the President appointed a committee, consisting of one delegate from each State, to nominate the regular Standing Committees of the Association.

The President announced Dr. Parsons, of R. I., as Chairman of the Committee appointed to memorialize Congress, relative to the importation of spurious and adulterated drugs, in the place of Dr. Sprague, resigned.

The Committee appointed to nominate the Standing Committees. were instructed also to report a suitable place for the holding of the next Annual Convention of the Association.

The reading of the reports of the Standing Committees was then resumed, and the report of the Committee on Medical Literature was read by Dr. Oliver W. Holmes, of Boston, Chairman of the Committee. The report embraced a retrospective and connected view and enumeration of the medical journals published in this country, with a synopsis of their most important contents.

The reports of the Committee on Obstretrics and Surgery were then taken up, with a view to a discussion of the merits of the use of chloroform or ether in these departments of medical science.

Dr. Warren, of Boston, spoke strongly in favor of these agents for removing pain, when administered with enlightened caution, and stated the results of some interesting experiments made by himself with an article which he styled chloric-ether, a modifiation of chloroform and ether, possessing many of the virtues of chloroform, without its dangerous properties.

At the conclusion of the remarks of Dr. Warren, the Association adjourned.

*Third day.*—The association was called to order at the usual hour, President Stephens in the Chair. The minutes of the last meeting having been read and accepted, Dr. Winne, of Baltimore offered a communication from the Medical Department of the National Institute, in reference to the appointment of a committee on Hygiene.

Dr. Roberts, from the committee of arrangements, reported additional members present. Also presented a statement of the United States Medical Corps, from members of the Navy, which was read. It related to what ought to be the qualifications of surgeons of the Navy, and was properly referred.

Dr. Cohen, of Baltimore, presented resolutions, complimentary to the surgeons of the Army and Navy, with a recommendation that a representation be made to Congress, and to the heads of the Medical Departments of Navy and War, expressing the gratification of the Association at the position recently assigned to the medical officers of the Navy, &c. The resolutions were adopted unanimously.

The committee to report a place of meeting for the next year, and the standing committees presented the result of their deliberations. They recommend the next place of meeting to be Boston., with a large number of gentlemen as standing comittees.

On the question of accepting that part of the report in relation to the place of meeting, considerable discussion arose. It was finally accepted, on the understanding that Cincinnati would be the place of meeting in 1850. The entire report was then accepted.

Dr. Wellford, of Fredericksburgh, Va. from the committee on medical education, read the report from that committee, which was accompanied by a number of resolutions..

On motion, the Association resolved itself into committee of the whole, and proceeded to the discussion of the resolutions, Dr. Knight, of Conn. in the chair.

The discussion was participated in by a large number of members, the principal question being upon the standard of education, and the granting of certificates to students, upon an examination after a first year's course of study. The committee, without coming to a conclusion, on motion of Dr. Stewart, of New York, rose, reported progress, and had leave to sit again.

Dr. Smith, of New York, Chairman of the committee on Practical Medicine, made a statement that he had unfortunately lost his trunk, containing his baggage, and with it the report, on his passage from Philadelphia. Every effort had been made to recover it, but unsuccessfully. He therefore could now but make a sketch of the heads of the report.

A resolution directing the chairman to furnish his report to the committee on publications was passed.

The Association again went into committee of the whole on the resolutions attached to the report on Medical Education, Dr. Knight in the chair. The discussion was an interesting one, and called out the most eminent members of the profession in this country. No two members seemed to have exactly the same views, on the subject of examination &c. but all seemed to think that some change was perhaps advisable. At two o'clock the committee rose, and the Association adjourned until four o'clock.

AFTERNOON SESSION.

The Association was called to order by the President.

A resolution to refer the communication from surgeons of the Navy to the Committee on Publications, directing it to be published among the proceedings, was offered and adopted.

Dr. Winne presented a resolution for the appointment of a committee of five, to take into consideration the communication of the Medical Department of the National Institute, on the subject of Hygiene, which was adopted and the committee appointed, with instruction to report before the close of the session.

Dr. Parsons, chairman of the committee to whom was referred the matter of memorializing Congress on the subject of the importation of deleterious drugs and chemicals, reported a memorial, which was adopted, with instructions that it be signed by the officers, and forwarded to the Hon. Dr. Thomas O. Edwards, for presentation to Congress.

The President handed in a report of the number of practitioners of medicine, so far as could be ascertained, who were acting without diplomas, which was referred to the committee on publications.

On motion the Association then resolved itself into a committee of the whole, and proceeded to the further discussion of the resolutions attached to the report of the committee on medical education, Dr. Knight in the chair.

The discussion was continued for a considerable time, various substitutes being offered, the last of which, offered by Dr. Thomas, of Baltimore, was adopted. This resolution was to the effect that the faculties of the several colleges be advised and requested to institute, daily or weekly examinations, recapitulatory of previous lectures, and to take such means as will secure the attendance of Students to the close of the term.

On the remaining resolutions, some discussion ensued, but the report was finally adopted, and the committee rose and reported it to the Association.

The report of the committee of the whole was then read, and adopted unanimously.

Dr. Atlee offered a resolution recommending the formation of State Medical Societies, where none now exist, which was adopted.

The committee appointed on the subject of Hygiene, reported in favor of appointing a committee of twelve on Hygiene, to report next year, which was adopted.

Propositions for the amendment of the constitution were made, and laid over until next year.

Several resolutions were offered and laid on the table.

Dr. Griscom, of N. Y. from the committee, reported in favor of a registration of births and deaths—report accepted, and referred to the committee on publications.

Dr. Power presented a resolution that the report of the committee on Medical Sciences, be referred to the committee on publications, which was adopted.

The resolution to appoint delegates to visit the Provincial and British Association was passed.

The report from the committee on Indigenious Botany, was referred to the committee on publications, and the committee continued for another year.

Several reports from Colleges &c. were referred to the committee on publications.

Dr. G. C. M. Roberts, Chairman of the Committee of Arrangements, submitted a report from which it appeared that the whole number of delegates accredited to the Association, was 477, of which number 277 were present, and the following States were represented, viz: New Hampshire, Vermont, Massachusetts, Rhode Island, Connecticut, New York, New Jersey, Pennsylvania, Delaware, Maryland, District of Columbia, Virginia, South Carolina, Louisiana, Texas, Georgia, Missouri, Indiana, Illinois, Kentucky, Tennessee, Ohio, and the Medical Corps of the Army and Navy of the United States.

On motion of Dr. Hamilton, the subject of the use of anaesthetic agents in surgery and obstetrics, was referred to the several standing committees with instruction to gather additional testimony in relation to their effects, and the safety or danger in their use. Adjourned.

*Fourth day.*—The association met this morning at 9 o'clock and was called to order by the President.

The President and Vice Presidents were authorized to appoint twelve delegates to represent the Association in the Provincial and the British Medical Associations of Great Britain.

On motion of Dr. Bouditch, of Boston, the thanks of the Association were tendered to the committee of Arrangements and of Reception, to the Faculties of the University of Maryland and of the Washington Medical

College, and to the members of the profession generally in Baltimore, for the hearty welcome and generous hospitality extended by them to the members of the Association.

The subject of the deleterious effects arising from the extensive consumption of tea and coffee by children and by the laboring classes, was referred on motion of Dr. Jackson, of the University of Pennsylvania, to the Committee on Public Hygiene.

Resolutions tendering the thanks of the Convention to the officers for the impartial discharge of their duties, and to the mayor of the city for courtesies extended, were adopted, and the Convention then adjourned *sine die*.

As the proceedings of the association will be published entire in a few weeks, with the reports of the standing and special committees, we do not deem it proper at present to make any remarks upon its doings, save to say that the meeting was characterized by general good feeling and harmony among the members, and that we have come away from this convention with more complete confidence in the conservative and progressive influence of the American Medical Association than we ever entertained before.

